# Supplementary material for: Assessment of Squalene-Adenosine Nanoparticles in Two Rodent Models of Cardiac Ischemia-Reperfusion
Source: Pharmaceutics. 2023 Jun 21;15(7):1790. doi: 10.3390/pharmaceutics15071790 (PMC10384353; doi:10.3390/pharmaceutics15071790)
Supplement: Supplementary file 1 [file pharmaceutics-15-01790-s001.zip › pharmaceutics-2370566-supplementary.pdf]

Supplementary data

Assessment of Squalene-Adenosine Nanoparticles in Two Rodent Models of Cardiac Ischemia-Reperfusion

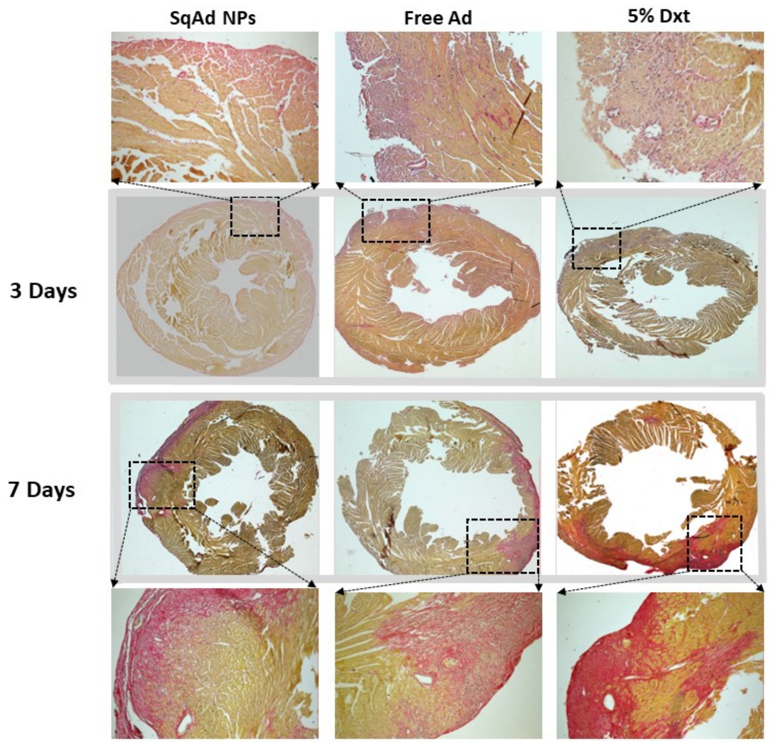

Figure S1: Picrosirius red staining revealed the presence of fibrosis on heart sections obtained from mice submitted to 30 minutes of ischemia and followed by 3- or 7-days of reperfusion. The inset shows the magnification of a selected area. Representative images of Picrosirius red staining (2.5 x; insets, 10x) for each group.

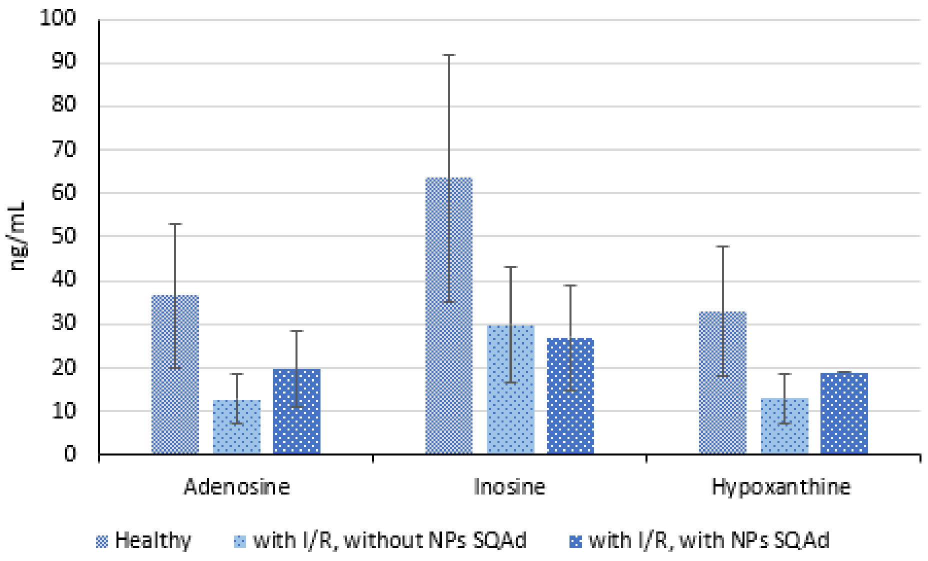

Figure S2: Whole blood quantification of adenosine, inosine and hypoxanthine.
250 µL of whole blood was taken from rats submitted to ischemia (30 min) and reperfusion (6 hours) and without prior administration of SQAd NPs. As control, 250 µL of whole blood was taken from healthy rats without prior administration of SQAd NPs.

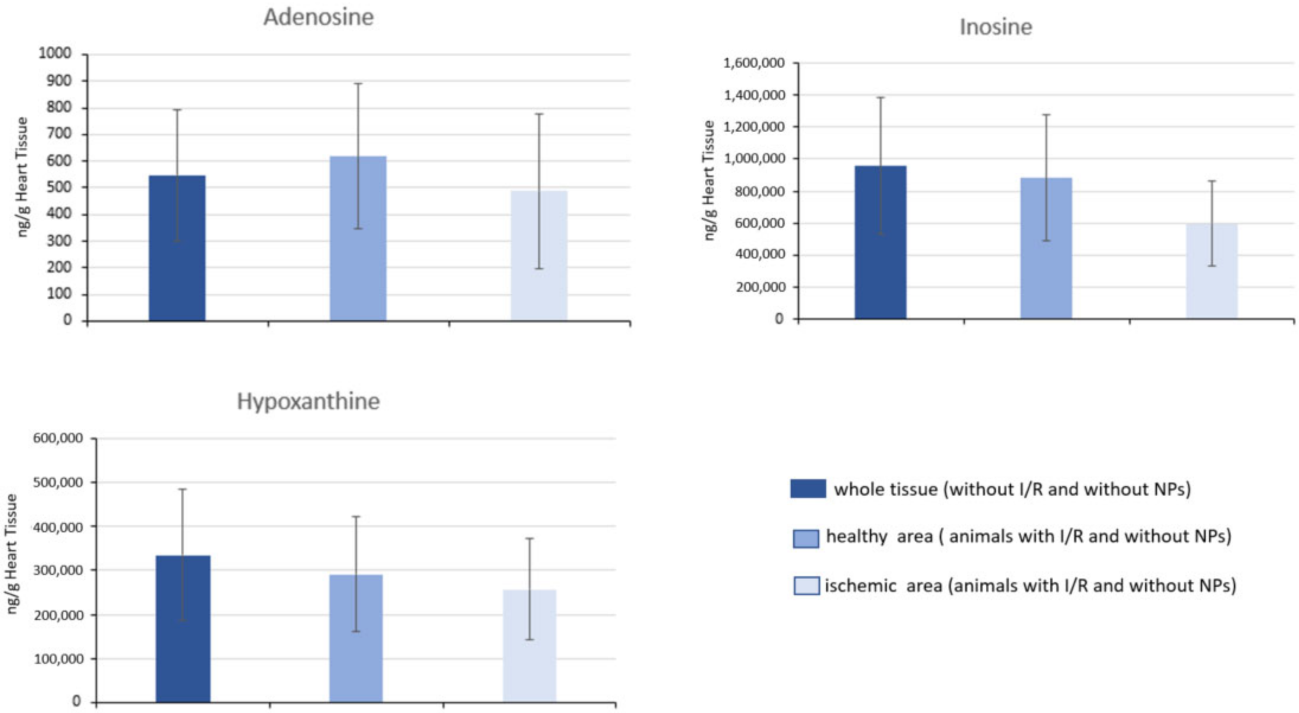

Figure S3: Cardiac quantification of adenosine, inosine and hypoxanthine.
Quantification was done on heart tissues from rats without or with ischemia (30 min) and reperfusion (6 hours) without prior administration of SQAd NPs. In rats with ischemia and reperfusion a comparison between ischemic and non-ischemic zone was performed.
